# Roles of Type 10 17β-Hydroxysteroid Dehydrogenase in Health and Disease

**DOI:** 10.3390/jpm15080346

**Published:** 2025-08-01

**Authors:** Xue-Ying He, Janusz Frackowiak, Song-Yu Yang

**Affiliations:** 1 Department of Molecular Biology, NYS Institute for Basic Research in Developmental Disabilities, Staten Island, NY 10314, USA; xue-ying.he@opwdd.ny.gov; 2 Department of Developmental Neurobiology, NYS Institute for Basic Research in Developmental Disabilities, Staten Island, NY 10314, USA; janusz.frackowiak@opwdd.ny.gov; 3 Ph.D. Program in Biology-Neuroscience, Graduate Center, The City University of New York, New York, NY 10016, USA

**Keywords:** ABAD, Alzheimer’s disease, cancer, neurosteroid metabolism

## Abstract

Type 10 17β-hydroxysteroid dehydrogenase (17β-HSD10) is the *HSD17B10* gene product. It plays an appreciable part in the carcinogenesis and pathogenesis of neurodegeneration, such as Alzheimer’s disease and infantile neurodegeneration. This mitochondrial, homo-tetrameric protein is a central hub in various metabolic pathways, e.g., branched-chain amino acid degradation and neurosteroid metabolism. It can bind to other proteins carrying out diverse physiological functions, e.g., tRNA maturation. It has also previously been proposed to be an Aβ-binding alcohol dehydrogenase (ABAD) or endoplasmic reticulum-associated Aβ-binding protein (ERAB), although those reports are controversial due to data analyses. For example, the reported *k*_m_ value of some substrate of ABAD/ERAB was five times higher than its natural solubility in the assay employed to measure *k*_m_. Regarding any reported “*one-site* competitive inhibition” of ABAD/ERAB by Aβ, the *k_i_* value estimations were likely impacted by non-physiological concentrations of 2-octanol at high concentrations of vehicle DMSO and, therefore, are likely artefactual. Certain data associated with ABAD/ERAB were found not reproducible, and multiple experimental approaches were undertaken under non-physiological conditions. In contrast, 17β-HSD10 studies prompted a conclusion that Aβ inhibited 17β-HSD10 activity, thus harming brain cells, replacing a prior supposition that “ABAD” mediates Aβ neurotoxicity. Furthermore, it is critical to find answers to the question as to why elevated levels of 17β-HSD10, in addition to Aβ and phosphorylated Tau, are present in the brains of AD patients and mouse AD models. Addressing this question will likely prompt better approaches to develop treatments for Alzheimer’s disease.

## 1. Introduction

**Figure 1:**
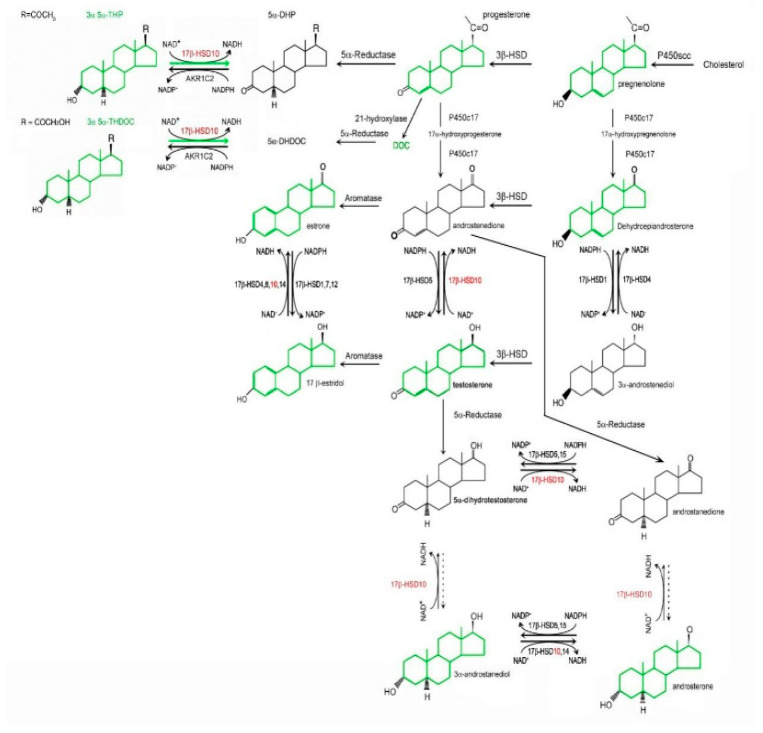
Roles of *17β-HSD10* in neurosteroid metabolism in maintaining homeostasis of allopregnanolone. *17β-HSD10* and neurosteroids are shown in red and green, respectively. Reproduced from Figure 3 of Ref. [1].

**Figure 2:**
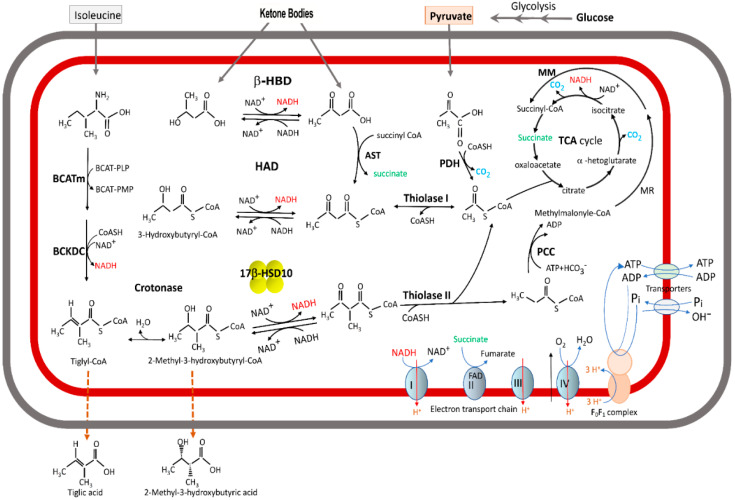
Roles of 17β-HSD10 in acyl thioester metabolism to generate ATP in brain mitochondria. Reproduced from Figure 2 of Ref. [1].

The *HSD17B10* gene was first cloned from the human brain (AF037438) and mapped to Xp11.2 in 1997 [1,2]. Its product, type 10 17β-hydroxysteroid dehydrogenase (17β-HSD10), is a mitochondrial, homo-tetrameric protein with a molecular weight of 108 kDa [1,2,3,4,5,6,7,8,9,10] (OMIM300256). This multifunctional protein is a central hub of vital enzymatic activities involved in different metabolic pathways, such as neurosteroid and hormone metabolism (see Figure 1) as well as fatty acid oxidation and branched-chain amino acid degradation [1,2,3,4,5,6,7,8,9,10,11,12,13,14,15,16,17,18,19,20,21,22,23,24,25,26] (see Figure 2). It is also capable of binding to other proteins whereby it participates in various physiological functions, e.g., 17β-HSD10 serves as a component of mitochondrial RNase P, namely MRPP2, necessary for tRNA maturation and mitochondrial integrity [23,24,25,26,27,28,29,30,31,32,33,34,35,36,37,38,39,40,41,42,43,44,45,46,47,48,49,50,51]. Studies on the properties of 17β-HSD10 are critical to the understanding and treatment of *HSD17B10* gene-related disorders [1,10,11,14,23,24,25,26,27,28,29,30,31,32,33,34,35,36,37,38,39,40,41,42,43,44,45,46,47,48,49,50,51,52,53,54,55,56,57,58,59,60,61,62,63,64,65,66,67,68,69,70,71,72,73,74,75,76,77,78], such as HSD10 mitochondrial disease (OMIM#300438), including HSD10 deficiency [9,19,21,22,23,24,25,26,27,28,29,30,31,32,33,34,35,36,37,38,39,40,41,42,43,44,45,46,47,48,49] and X-linked mental retardation; choreoathetosis and abnormal behavior (MRXS10, OMIM#300220) [60,61] resulting from a missense and silent mutation on the *HSD17B10* gene, respectively; and Alzheimer’s disease (OMIM#104310), where elevated levels of 17β-HSD10 found in brain cells (see Figure 2 of Ref. [65]) [1,2,3,4,5,6,14,21], and to the elucidation of its protection in Parkinson’s disease [62,63]. Because the *HSD17B10* gene product has various functions and is implicated in the pathogenesis of many diseases, including hormone secretion-related cancers [64,65,66] and diabetic kidney disease [67], it has received at least ten alternative designations (see Table 1).

## 2. What Are ERAB and ABAD?

Since ERAB and ABAD were listed as alternative terms of 17β-HSD 10 in the OMIM 300256, people need to know what ERAB and ABAD are. ERAB (*endoplasmic reticulum*-associated binding protein), an artificial 27 kDa peptide [51,52,53,54,55], was later renamed ABAD (Aβ-binding alcohol dehydrogenase) [56,57,58], following the establishment of 17β-HSD10 as a *mitochondrial* protein due to an N-terminal mitochondrial-targeting signal (see Figure 1 of Ref. [1]) [1,6,9,10,11,12,13]. The ERAB [51,52,53,54,55] and ABAD [56,57,58,59] are thus both *outdated* alternative terms of, or misnomers for, 17β-HSD10 [1,6,9,10,11,12,13,21] (see Table 1 and OMIM300256).

Reports of ERAB/ABAD in high-impact journals [51,52,53,54,55,56,57,58] have not been subject to a requirement for published corrigenda by the editors of those journals, although those editors have long been informed of the ABAD/ERAB work that necessitates such corrigenda [3,11,12,13,21].

17β-HSD10 is a protein necessary for life. It is found in all tissues and is most abundant in the liver and brain (see the human protein atlas—HSD17B10). Its relative levels in various brain regions are shown in Figure 2 of Ref. [69].

### 2.1. ERAB Is an Artificial Protein That Is Not Present in Any Tissues

**Figure 3:**
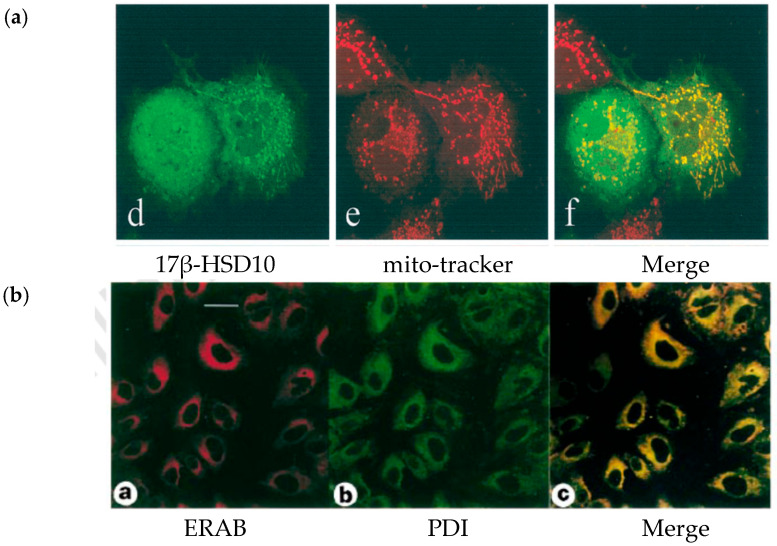
Comparison of the reported intracellular localization data of 17β-HSD10 (**a**) with those reported for ERAB (**b**). The 1st column in (**a**,**b**) showed the staining of corresponding protein; the 2nd column in (**a**,**b**) showed the staining of mitochondria and the endoplasmic reticulum (ER), respectively. The 3rd column in (**a**,**b**) shows the merge of the images of the 1st and 2nd columns in the same row. a and b were reproduced from Figure 1 of Ref. [6] and Figure 6 of Ref. [51], respectively.

According to a report in a high-impact journal [51], ERAB consists of 26***2*** amino acid residues and is associated with the *endoplasmic reticulum*. But such an ERAB has never been isolated from human tissues. Instead, it was found [1,6,7,8,9,10,11,12,13,14,15] that the *HSD17B10* gene encodes a multifunctional enzyme 17β-HSD10/SCHAD/HADII primarily located in the *mitochondria* (see Figure 3a). A *subunit* of this mitochondrial 17β-HSD10 homo-tetramer consists of 26**1** amino acid residues and has an *almost* identical amino acid sequence to ERAB [1,2,3]. The ERAB was reportedly associated with the ER in high-impact journals [51,52,53,54]. It is now established that ERAB is only an *artificial* protein, and it was *not* known whether it showed any catalytic activity since its reported activities [54] were non-reproducible [11]. As is well known, most proteins are synthesized in the endoplasmic reticulum and then migrate to other specific destinations. Thus, the concept of an “ERAB” as a **27 kDa peptide *associated with the endoplasmic reticulum that plays a key role in the pathogenesis of AD*** [51,52,53,54,55] is patently erroneous [1,2,3,9,10,11]. Surprisingly, in light of these unambiguous findings, the ERAB authors never published any corrigendum nor have they withdrawn such egregiously erroneous reports [51,52,53,54,55] but rather renamed ERAB as ABAD in a subsequent report in the high-impact journal *Science* [58]. The journal *Nature* generally rapidly responds to readers’ challenges [73], but *Nature* has been inexplicably silent in this matter. Further, the ERAB/ABAD authors never explained why their ERAB findings had to be renamed ABAD and why ABAD was *also* reported to be associated with the ER according to earlier ABAD reports [54]. These authors should clarify the distinction between ABAD [54,56,58,59] and ERAB [51,52,53,55] for the scientific community, particularly where some ABAD/ERAB researchers have begun to refer to ABAD/ERAB as 17β-HSD10 [74].

### 2.2. 17β-HSD10 Is a Member of the Alcohol Dehydrogenase Family

Characterization of the alcohol dehydrogenase and hydroxysteroid dehydrogenase activities of type 10 17β-hydroxysteroid dehydrogenase (17β-HSD10)/short-chain 3-hydroxyacyl-CoA dehydrogenase (SCHAD) has been reported since the year 1999 [3]. This multifunctional enzyme was found to be involved in fatty acid oxidation and the metabolism of neurosteroids, ketone bodies, and isoleucine [2,3,4,5,6,7,8,9,10,11,12,13,14,15,16,17,18] (see Figure 1 and Figure 2). The enzyme 17β-HSD10/SCHAD [6,7] is apparently a new member of the alcohol dehydrogenase family [1,13,72,73,74,75,76,77,78,79,80,81,82,83] and distinct from the classical short chain 3-hydroxyacyl-CoA dehydrogenase [84]. In contrast, the reported “generalized” alcohol dehydrogenase activities of ABAD (see Table 2) were subsequently established to be erroneous.

Since ABAD and 17β-HSD10 were considered identical proteins, the moniker ABAD/ERAB is an *outdated* alternative term for 17β-HSD10/SCHAD [64] (see Table 1 and OMIM300256:17β-Hydroxysteroid dehydrogenase type X). However, by comparing their reported catalytic functions, it was surprisingly found that the measurement of ABAD/ERAB’s enzyme catalytic functions performed according to the reported experimental procedures [60] could not have been obtained by any valid data (see Table 2). It is also not known why the ERAB/ABAD authors developed a purportedly “*unique* enzyme assay” method, because the L-3-hydroxyacyl-CoA dehydrogenase assay had already been published and the *Methods of Enzymology* [77].

The term endoplasmic reticulum-associated Aβ-binding protein (ERAB) [58] was apparently reluctantly changed to ABAD (Abeta-associated alcohol dehydrogenase) in 2000 [54], since 17β-HSD10/SCHAD had reportedly been localized in mitochondria rather than the ER [2,3,6], although ERAB was reported to have a non-cleavable ER signal peptide [55]. Furthermore, it is not understandable why *no* 17β-HSD10/SCHAD literature appeared as a reference in the ABAD *Science* report [58], particularly where some of such references, e.g., Ref. [2] here, had once been cited in an ERAB article [53] as reference 21. We hope related journals will pay some attention to this problem and provide an explanation in future reports and/or in corrigenda, especially because a recent special issue of the *Science* journal emphasized *research integrity*. This is particularly significant where it is implausible that the publishers had or have *no* knowledge about a dozen 17β-HSD10/SCHAD publications in well-established journals, e.g., Refs. [6,7,8,12,13,79,80]. This abject failure to cite relevant literature was repeated in a recent review of ABAD [57], where *no* 17β-HSD10/SCHAD literature was cited. More absurdly, ABAD was recently referred to as “the complex of Aβ/ADH,” [57] in spite of the availability of a rich background literature establishing the contrary.

Since the Human Genome Organization (HUGO) had announced the official term of this gene to be *HSD17B10* (see the footnote in Table 1), the proper name of the gene product is established as 17β-hydroxysteroid dehydrogenase type 10 (see NIH/OMIM 300256). According to the “**instruction to authors**” of all related journals, it would appear inappropriate to use the out-of-date moniker ABAD rather than the official term 17β-HSD10 for the title of a *scientific report* [56,57,59] to avoid serious confusion to readers.

### 2.3. From 2-Methyl-3-Hydroxybutryl-CoA Dehydrogenase (MHBD) to 17β-HSD10

In 1995, a new type of 3-hydroxyacyl-CoA dehydrogenase, namely HADII, was isolated from rat liver mitochondria [75]. This enzyme is a member of the short-chain dehydrogenase/reductase (SDR) family [79]. It can catalyze the β-oxidation of branched-chain fatty acids, e.g., 2-methyl-3-hydroxybutyryl-CoA (see Figure 1), so it was also labeled as 2-methyl-3-hydroxybutyryl-CoA dehydrogenase (MHBD) [4,7,9,10,11,12,13,14,15,16,17,18,35,36,37,38,39,40,41,42,43,44,45,46,47,48,49,50,51,52,53,54,55,56,57]. Human MHBD was then found to be a multifunctional enzyme catalyzing the metabolism of neurosteroids, including allopregnanolone, a steroid modulator of the γ-aminobutyric acid type A receptor (see Figure 2 of Ref. [18]) [18,19,20,21,22,23,24,25]. Since this multifunctional enzyme plays a key role in the regulation of neuronal excitability, it was eventually designated as type 10 17β-hydroxysteroid dehydrogenase (17β-HSD10) by the International 17β-hydroxysteroid dehydrogenases workshop [14]. A missense and silent mutation on the human 17β-HSD10 gene results in 2-methyl-3-hydroxybutyryl-CoA dehydrogenase/HSD10 deficiency with developmental disabilities [32,33,34,35,36,37,38,39,40,41,42,43,44,45,46,47,48,49] and mental retardation, choreoathetosis, and abnormal behavior (MRXS10) [60,61], respectively.

### 2.4. Short-Chain/Medium-Chain 3-Hydroxyacyl-CoA Dehydrogenase Deficiency Is Distinct from MHBD/HADHII/HSD10 Deficiency

Short-chain/medium-chain 3-hydroxyacyl-CoA dehydrogenase deficiency is a metabolic inborn error in the fatty acid β-oxidation pathway (see Figure 1 of Ref. [81]) due to the lack of L-3-hydroxyacyl-CoA dehydrogenase activity, resulting in the accumulation of acetoacetic acid [82,83]. It was named by some clinicians as short-chain 3-hydroxyacyl-CoA dehydrogenase (SCHAD) deficiency [83], but this inherent metabolic disorder is certainly distinct from HSD10 deficiency, even though 17β-HSD10 had an alternative moniker “SCHAD” in the previous century [2,3].

### 2.5. Aβ Binds 17β-HSD10 to Inhibit Its Enzyme Activity

A popular proposition [51,52,53,54,55,56,57,58,59] that ERAB mediates Aβ neurotoxicity to destroy neurons was established to be erroneous [1,4,10,72] although those ABAD/ERAB studies have been funded for tens of millions dollors. In reality, Aβ binds with 17β-HSD10 to inhibit its enzymatic activities [1,4,71], resulting in damage to brain cells.

## 3. Kinetic Constants of ABAD/ERAB Not Derived from Experiments

ERAB/HADH II was studied for its activity to reduce *S*-acetoacetyl-CoA, as well as its capacity to dehydrogenate alcohol groups in a range of linear alcohols and in sterols such as 17β-estradiol [1,2,3,9,10,21,72]. The experimental procedures employed for the determination of ERAB/ABAD enzymatic activities are quoted from the reference [53] as follows:

*‘The assay for reduction of S-acetoacetyl-CoA employed ERAB/HADH II (333 ng/mL), a range of S-acetoacetyl-CoA concentrations (0.0015–0.36 mM; Sigma, St. Louis MO, USA), and NADH (0.1 mM; Sigma) in 97 mM potassium phosphate (pH 7.3). The reaction was run for a total of 2 h at 25 °C under steady-state conditions (34)^†^, and the change in NADH absorbance at 340 nm was determined every 5 min.’*

*‘Alcohol dehydrogenase assays employed ERAB/HADH II (20 μg/mL), a range of alcohol substrates and concentrations (methanol, ethanol, n-propanol, isopropanol, n-butanol, isobutanol, n-pentanol, (±)-2-octanol, (+)-2-octanol, (−)-2-octanol, and n-decanol; Sigma), and NAD^+^ (7.5 mM) in 22 mM sodium pyrophosphate, 0.3 mM sodium phosphate (pH 8.8). The reaction was run for 2 h at 25 °C, and the absorbance at 340 nm was monitored every 5 min as described above.’*

Because 3-hydroxyacyl-CoA dehydrogenase (HAD) was the first enzymatic activity found in the *HSD17B10* gene product, human 17β-HSD10 was then designated as short-chain 3-hydroxyacyl-CoA dehydrogenase (SCHAD) [2,3]. Research on the reduction of S-acetoacetyl-CoA by NADH catalyzed by ERAB/ABAD was also reported (see Table 1, Figure 2A and Figure 5A of Ref. [53]). Unfortunately, it was found [11] that the reported kinetic constants of ERAB/ABAD are *not* based upon assays performed under the reported experimental conditions, where ΔA_340_ (the absorbance change at 340 nm) was determined every 5 min until 120 min and the coenzyme added was 0.1 mM NADH. Although the concentrations of substrate (S-acetoacetyl-CoA) used for such assays were reportedly from 0.0015 mM to 0.36 mM, the enzymatic reaction would indeed stop after 0.1 mM substrate was used up, while the coenzyme 0.1 mM NADH had been oxidatively exhausted. The reaction was reportedly catalyzed by ERAB (333ng/mL), so the enzymatic reaction would already be over before 5 min—the first data point allegedly observed—if the reported kinetic constants (V_max_, K_m_) [53] are taken for granted.

**Figure 4:**
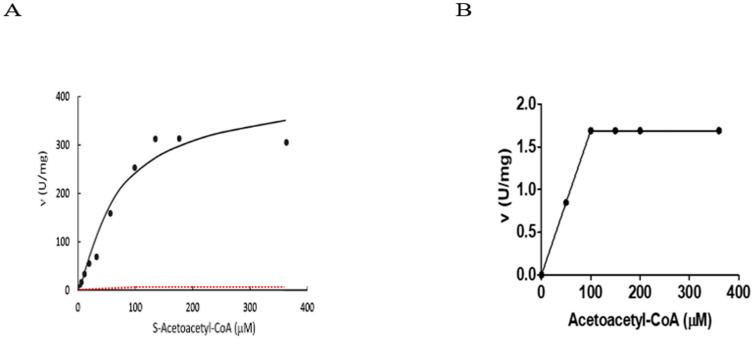
Reduction of acetoacetyl-CoA catalyzed by 17βHSD10/ERAB. (**A**) Reproduced from Figure 4 of Ref. [4]; (**B**) reproduced from the bottom of Part A by magnifying the ordinate of a red dotted line shown in Part A.

Assuming the data are collected by real use of the experimental procedure reported in Ref. [53], a plot of ΔA_340_/min versus [S-acetoacetyl-CoA] could be achieved as shown in Figure 4B*. It appears to be a refraction line with a turning point at 0.1 mM S-acetoacetyl-CoA rather than a previously reported *hyperbola* fitting with the Michealis–Menten equation [85,86] (see Figure 2A of Ref. [57]). It was demonstrated that the assay designers appeared to lack *basic* biochemical knowledge, such as the essential concept of *initial velocity* (*v*) [84], but a much more severe problem was the *inconsistency* between the published *data* and *experimental procedures,* regardless of whether those reported procedures were scientifically appropriate (see Figure 4). A comparison of actual kinetic constants of the *HSD17B10* gene product [6,9,12] to those reported for ERAB/ABAD [53] was already reported [11,12]. The abstract of that article shown in the *Biochemical Journal* is as follows:

*‘The alcohol dehydrogenase (ADH) activity of human short-chain L-3-hydroxyacyl-CoA dehydrogenase (SCHAD) has been characterized kinetically. The k_(cat)_ of the purified enzyme was estimated to be 2.2 min^−1^, with apparent K(m) values of 280 mM and 22 mM for 2-propanol and NAD^+^, respectively. The* *k_cat_ of the ADH activity was three orders of magnitude less than the L3-hydroxyacyl-CoA dehydrogenase activity but was comparable with that of the enzyme’s hydroxysteroid dehydrogenase (HSD) activity for oxidizing 17beta-oestradiol [He, Merz, Mehta, Schulz and Yang (1999) J. Biol. Chem. 274, 15014-15019]. However, the k_cat_ values of intrinsic ADH and HSD activities of human SCHAD were found to be two orders of magnitude less than those reported for endoplasmic-reticulum-associated amyloid beta-peptide-binding protein (ERAB) [Yan, Shi, Zhu, Fu, Zhu, Zhu, *et al. *(1999) J. Biol. Chem. 274, 2145-2156].*

*Since human SCHAD and ERAB apparently possess identical amino acid sequences, their catalytic properties should be identical. The recombinant SCHAD has been confirmed to be the right gene product and not a mutant variant. Steady-state kinetic measurements and quantitative analyses reveal that assay conditions such as pH and concentrations of coenzyme and substrate do not account for the kinetic differences reported for ERAB and SCHAD. Rather problematic experimental procedures appear to be responsible for the unrealistically high catalytic rate constants of ERAB. Eliminating the confusion surrounding the catalytic properties of this important multifunctional enzyme paves the way for exploring its role(s) in the pathogenesis of Alzheimer’s disease.’*

The reported catalytic capability of ABAD/ERAB [53] was found to be unfortunately greatly exaggerated by ABAD/ERAB researchers at will [1,4]. It was also found [1,4] that the ABAD data reported in another *JBC* article [54] were impossible to reproduce.

## 4. Re-Discovery of ABAD/ERAB in Mitochondria

After nucleotide sequences of this gene (*HSD17B10* AF037438) and its cDNA (AF035555) were deposited into the GenBank in 1997 [2], an article appeared in *Nature* in which a 27kDa Aβ-binding protein with ***262*** amino acid residues was reportedly to be associated with the endoplasmic reticulum, namely the endoplasmic reticulum-associated Aβ-binding protein (ERAB) [51]. Since the *HSD17B10* gene product (17β-HSD10/SCHAD) was isolated and demonstrated to be a mitochondrial homo-tetrameric protein of which each subunit consists of **261** amino acid residues [2,3,6,7,8,9,10,11,12,13,14,15,18,21], ERAB was compelled to be *redesignated* as Aβ-binding alcohol dehydrogenase (ABAD) based on its so-called *generalized alcohol dehydrogenase activities (C2-C10)* [53,54]. Furthermore, the intracellular localization of ABAD and ERAB (see Figures 6–8 of Ref. [53] and Figure 2c of Ref. [54]) were later changed from ER [51,52,53,54] to mitochondria and then published in *Science* as a new discovery [58] by *omitting* all earlier literature establishing that the intracellular localization of *HSD17B10* gene product is in the mitochondria [2,3,9,10,11,12,13,81,82,87], because it carries an N-terminal mitochondrial targeting signal [1,2,3,4,5,6,7,8,9,10,11,12]. More surprisingly, no explanations for the following inconsistencies have ever been provided by those high-impact journals [58,59,60,61,62,63,64,65], e.g., why immune-histological micrographs stained with guinea pig or mouse anti-ABAD [65] did not resemble those stained with rabbit anti-ERAB/ABAD [51,52,53,54,55,56,57,58]. In particular, Figure 1 of Ref. [65] could be questioned as to whether it could replace Figure 4 of Ref. [58] published also by Yan S. et al., since ERAB and ABAD are just alternate names for the *HSD17B10* gene product, namely 17β-HSD10/SCHAD [6,7,8,9,10,11,12,13,14,15,16,17,18]. Ref. [6] here had been cited once in the Ref. [53] as its Ref. [21], so *both ABAD’s authors and reviewers should already be aware of 17β-HSD10/SCHAD studies.* Intentional omission of key literature [2,3,11,12,13] in that ABAD *Science* article [58] did nothing to help the credibility of those ABAD/ERAB reports and exhibited no consideration for the integrity of published scientific research.

## 5. Can Competitive Inhibition Be Defined by a Single Concentration of Substrate?

Investigation of the inhibition of the *HSD17B10* gene product, 17β-HSD10, by Aβ is underway. It was reported to be a *one-site competitive inhibition* [53], and a deputy editor of *JBC* had lately created an equation for calculating the value of K_i_ (see the ASBMB letter in the SM2 of Ref. [6]). However, it is evident that only a single concentration of substrate and coenzyme, i.e., 0.18 mM S-acetoacetyl-CoA and 0.1 mM NADH, was used in that study (see the legend for Figure 5 of Ref. [53]) where an ordered Bi-Bi reaction proceeds. As a result, there is no reason to believe that the reported inhibition [53] had been demonstrated to be competitive inhibition.

Since it was reported [10] that the Hill coefficient of a 17β-HSD10 mutant is 1.3, it could not be expected that there is no interaction between subunits of this dehydrogenase. In other words, the rate equation provided by the editor of *JBC* may not be useful, since the rate equation presented by the *JBC* editorial board is only applicable to a monomer or a polymer without any interactions between its four active sites and not behaving as an allosteric enzyme [86]. In short, the rate equation for the reported *one-site competitive inhibition* [53] is still missing, and the *JBC* editorial board still needs to provide a proper answer to the scientific community.

## 6. What Is the Scientific Basis to Designate a So-Called “Aβ-Binding Alcohol Dehydrogenase”?

### 6.1. How Does the Aβ-Binding Alter the 3D Structure of 17β-HSD10?

**Figure 5:**
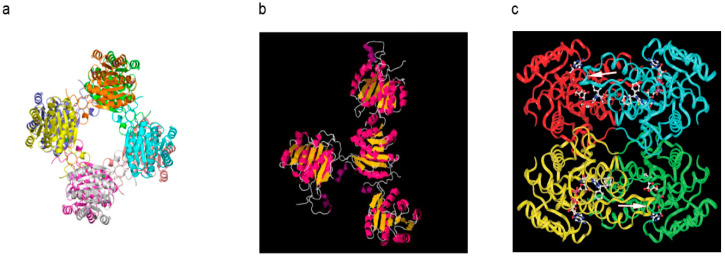
A comparison between three-dimensional structures of Aβ-bound ABAD [58] (**a**,**b**) and that of 17β-HSD10/ABAD [7] (**c**).

Information about the three-dimensional structure of this protein was available [7] (PDB1U7T, see Figure 5c). It is uncertain whether the binding of Aβ would lead to radical changes from what was seen in Figure 5c to that in Figure 4b (PDB1S08) because such a dramatic increase in the protein’s surface would need a supply of much free energy, and such a high energy status is certainly much more unstable. Since no electron density of Aβ was displayed in that *Science* article [58] and there is a clear difference between Figure 4a and Figure 4b that lacks an explanation [58], the reliability of the reported Aβ-bound ERAB/ABAD structure is questionable. In addition, whether there is a large solvent channel in the center of ABAD [58] (see Figure 5a) as compared with the 17β-HSD10 [7] (Figure 5c) needs to be clarified by further investigation.

### 6.2. Reported Alcohol Dehydrogenase Activity Data of ABAD Are Non-Reproducible

**Figure 6:**
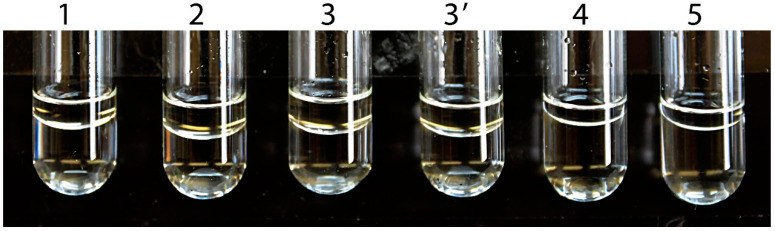
Aβ-binding alcohol dehydrogenase assay mixture prepared according to the experimental procedure shown in Ref. [53]. There is an interface between the two layers of the assay mixture, so it is not feasible to determine ABAD activity spectrophotometrically as had been reportedly accomplished by Yan SD et al. [53]. Reproduced from Figure 8 of Ref. [1].

We have questioned the reliability of ABAD/ERAB data shown in various journals since the year 2000 [11]. The related high-impact journals have ignored such challenges. For example, another *JBC* editor also argued by a phone call that since 1% DMSO was added to the assay system, it would solubilize all long-chain alcohols. Here, actual experimental data show that this argument is not true (see Figure 6).

It is well-known that alcohols with an alkyl > 6 carbon have poor solubility. (-)-2-octanol, (+)-2-octanol, and (±)-2-octanol are oils at 25 °C (see Windholz M (1983) *The Merk Index* 10th Edition, Merk & Co., Inc.) Since the solubility of (−)-2-octanol and (+)-2-octanol is only 6 mM and 8.5 mM, respectively, the solubility of racemic (±)-2-octanol could not be greater than 15 mM. The editor of that high-impact journal should have suspected the accuracy of the reported K_m_ values of ABAD/ERAB for (−)-2-octanol, (+)-2-octanol, and (±)-2-octanol to be 43, 84, and 85 mM, respectively. As shown in Figure 6 and Table 2, it is not feasible to determine ABAD activity towards 2-octanols using a spectrophotometer by following the published experimental procedures [53]. Obviously, no reliable data support the claim that the *HSD17B10* gene product exhibits *generalized* alcohol dehydrogenase activities (C2–C10), which underlies the conception of ABAD [54,55,56,57,58] that is the subject of a *Science* report [58] and, thereafter, dozens of ABAD reports published in various journals until the present time [57]. The term ABAD or ERAB originated most probably from a lack of basic chemical and biochemical knowledge, and the term ABAD/ERAB should be abandoned for 17β-HSD10 without exception.

Changes in 17β-HSD10 levels in brain cells [1,2,3,69] or mutations at the *HSD17B10* gene [32,33,34,35,36,37,38,39,40,41,42,43,44,45,46,47,48,49], in addition to elevated Aβ and phosphorylated Tau, can result in neurodegeneration (see OMIM#300438). As non-erroneous information prevails in this research field, we anticipate that more contributions will be made to the understanding and treatment of neurodegeneration [24,25,26,27,28,29,30,31,32,33,34,35,36,37,38,39,40,41,42,43,44,45,46,47,48,49,50,51,52,53,54,55,56,57,58,59,60,61,62,63,64,65,66,67,68,69,70,71,72,73,74,87,88,89,90,91,92,93,94,95,96,97,98,99].

## 7. Roles of 17β-HSD10 in Neurosteroidogenesis

**Figure 7:**
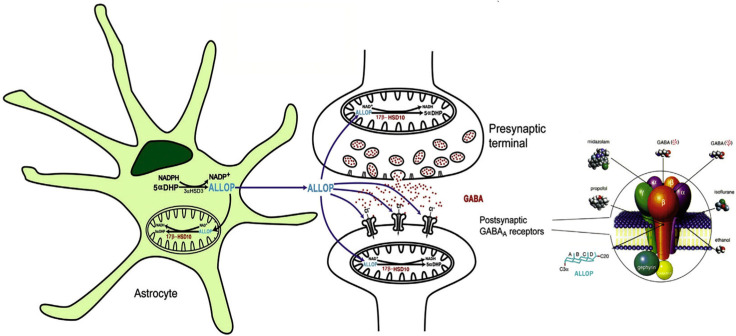
Homeostasis of allopregnanolone (ALLOP) in brain cells maintained by a dual enzyme molecular switch consisting of 17β-HSD10 and 3ɑ-HSD3(AKR1C2). The binding sites of individual modulators on the GABA_A_ receptor are shown in the circle on the right side. Reproduced from Figure 4 of Ref. [1].

Neurosteroids are important regulators of neuronal excitability and can be synthesized in mitochondria where 17β-HSD10 and cytochrome P450 enzymes are localized. As shown in Figure 7, the homeostasis of allopregnanolone (ALLOP), a positive modulator of GABA_A_ receptor, potentiates GABA to increase the opening of Cl^−^ channels. Normal excitability of brain cells is maintained by a *dual enzyme molecular switch* consisting of 17β-HSD10 and 3ɑ-HSD3 (AKR1C2). In contrast to the 17β-HSD10 studies, ABAD/ERAB researchers have never paid any attention to the important roles of this mitochondrial protein in either acyl thioester [1,2,24] or neurosteroid [1,13,15] metabolism. They are not aware that this is a multifunctional protein critical to maintaining neuronal excitability by a dual enzyme molecular switch [21].

## 8. Mitochondrial 17β-HSD10 Associated with ER by a “Modified” Cell Fractionation

It is not conceivable how ERAB/ABAD reports could purport to present data attempting to establish that a mitochondrial protein is associated with the ER [51,52,53,54,55,56,57,58,59,60,61]. In addition to reporting unverifiable immunocytochemical data (see Figure 3b,c), these reports also used an allegedly *novel* cell fractionation procedure, even though they reportedly cited a paper [100] as the methodology basis of the “*modified*” cell fractionation. Nevertheless, that cited paper [100] did not describe any details concerning its methodology. The details of so-called *modified* cell fractionation were revealed by late Nobel laureate Prof. C. de Duve, the pioneer of the *traditional* cell fractionation method. He pointed out that it is inappropriate to apply *cytochrome C* rather than *cytochrome C oxidase* as the mitochondrial marker in so-called *modified* subcellular fractionation procedure because this could lead to a mitochondrial protein being erroneously classified as an ER-associated one because cytochrome C would drop during cellular fractionation (see SM1 of ref. [6]).

**Figure 8:**
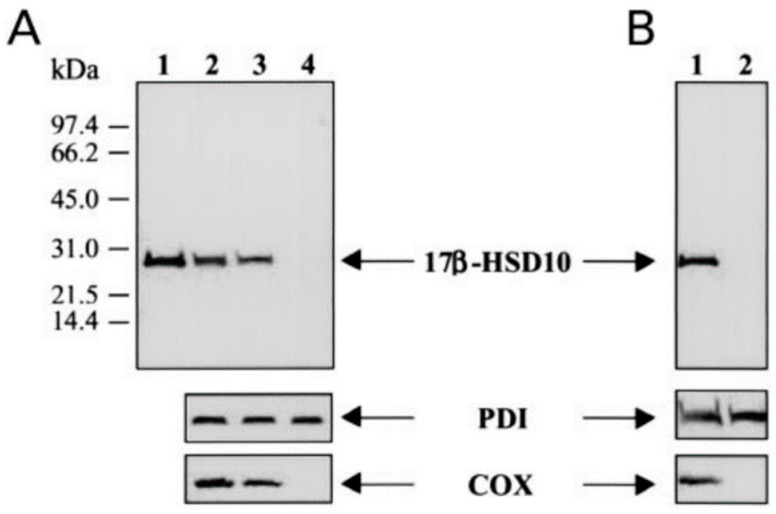
Comparison of subcellular localization of 17-HSD10 determined by conventional [9,10,11,12,13] and modified [51,52,53,54,55,56,57,58] fractionation procedures. (**A**) Purified human 17β-HSD10 (0.2 μg) (lane 1), homogenate (lane 2), pellet (lane 3), and supernatant (lane 4) were immunoblotted using rabbit anti-17βHSD10 antibody (top), mouse monoclonal anti-PDI Ig (middle), and anti-COX (bottom). (**B**) The ER fraction obtained from the modified (lane 1) and conventional protocol (lane 2) was immunoblotted using rabbit anti-17β-HSD10 Ig (top), mouse monoclonal anti-PDI Ig (middle), and anti-COX antibody (bottom). Reproduced from Figure 5 of Ref. [13].

Although those erroneous *JBC* reports had been cited in various journals for two decades, the scientific community—both researchers and clinicians—are still waiting for the necessary corrigenda of such manipulations in ERAB/ABAD studies [65,66,67,68,69,70,71,72]. Apparently, the so-called *modified* method shown in those ERAB articles [65,66,67,68] shall no longer be used in biomedical studies, even if they were published in high-impact journals (see Figure 8).

How could the ERAB/ABAD researchers perform immuno-neurochemical studies to confirm their erroneous subcellular fractionation “data” [51,52,53,54,55,56,57,58]? Related editors need to provide answers to the readers of their journals.

If the traditional method were used in such a study, 17β-HSD10 would have been proven to be a mitochondrial protein (see Figure 8). It is apparent to us [1,2,3,9,10,11,12,13,14,21] that the suspicious ERAB/ABAD data [51,52,53,54,55,56,57,58] were obtained erroneously.

## 9. Neurodegeneration Results from Qualitative or Quantitative Alteration of 17β-HSD10

It was reported [101] that Aβ was absent in the CSF of patients suffering infantile neurodegeneration. This finding indicates that the pathogenic role of 17β-HSD10 in neurodegeneration is not limited to its so-called “mediation of Aβ neurotoxicity.” Reduced expression of 17β-HSD10 was also considered to result in the pathogenesis of X-linked mental retardation, choreoathetosis, and abnormal behavior (MRXS10) [60,61]. Apparently, not only 17β-HSD10 mutants but also abnormal levels of this protein would cause neurodegeneration. Nevertheless, the Aβ binding of ERAB/ABAD reported by ABAD/ERAB researchers [51,52,53,54,55,56,57,58] had been revealed just to be one of the factors affecting the 17β-HSD10 activity. To keep a normal 17β-HSD10 activity is essential to the maintenance of mitochondrial function and structure [1,2,3,4,5,6,7,8,9,10,11,12,13,14].

## 10. Concluding Remarks

Research studies on the *HSD17B10* gene product have been severely compromised for almost two decades by the ABAD data that are demonstrated here to be irreproducible. It is fortunate that the published 17βHSD10 catalytic properties can be used to replace the erroneous data of the human ABAD studies to avoid further damage resulting from such erroneous ABAD reports. If the involved journals were obliged to publish corrigenda, it would encourage scientists to seek answers to the important question as to why elevated levels of 17β-HSD10 besides Aβ and phosphorylated Tau are present in the brains of AD patients and mouse AD models. It may open a new approach to the understanding and finding of good treatments for Alzheimer’s disease.

## Figures and Tables

**Table 1:** Alternative designations of human *HSD17B10* gene product ^‡^.

Year	Accession Number		Name	Acronym	Comments	Ref.
	cDNA	Gene				
1997–1998	AF035555 AF037438 [11/21/97] [12/9/97] Deposited into the Genbank		Short-chain 3-hydroxyacyl-CoA dehydrogenase	*SCHAD*	MW = 108 kDa, composed of 1044 residues. Homotetrameric enzyme exhibited HAD and 17β-HSD activity proposed to reside in mitochondria	[2]
	U96132 n/a		Endoplasmic reticulum-associated Aβ-binding protein	ERAB	MW = 27 kDa, composed of 262 amino acid residues and associated with the endoplasmic reticulum	[51,52,53]
1999			Novel 17β-Hydroxysteroid dehydrogenase	*Novel 17β-HSD*	Mitochondrial, multifunctional protein inactivates 17β-estradiol to estrone	[3,12]
			Amyloid β-peptide binding alcohol dehydrogenase	ABAD	Substitution of ABAD to ERAB but maintains association with ER and further conveys incongruous data of *generalized* alcohol dehydrogenase (C2-C10) activities	[54]
2000	AF035555 AF037438		2-methyl-3-hydroxyacyl-CoA dehydrogenase	MHBD	This term is appropriate especially for isoleucine metabolism	[31,32,46,47,48,76]
2001	OMIM300256: 17β -Hydroxysteroid dehydrogenase X		Type 10 17β-Hydroxysteroid dehydrogenase	*17β-HSD10*	Involved in neurosteroid, such as allopregnanolone, metabolism, and identification of its N-terminal mitochondrial-targeting signal	[1,2,3,4,10,11,45]
2004			Amyloid β-peptide binding alcohol dehydrogenase	ABAD	Renames ER-associated ABAD to be a mitochondrial ABAD but still ignores reported activities of 17β-HSD10	[58]
2007	NM_004493, Gene symbol: *HSD17B10* *		3-Hydroxyacyl-CoA dehydro-genase type 2	HADH2	A silent mutation was found in MRXS10 ** patients	[61]
2008			Mitochondrial RNase P protein 2	MRPP2	A component of the RNA-free RNase P complex	[23,24,25,26]
2015			Short-chain de- hydrogenase/re-ductase 5C1	SDR5C1	Shown in a short-chain dehydrogenase/reductase (SDR) evolution tree	[30]

^‡^ Updated from Table 1 of Ref. [1]; * This official gene name *HSD17B10* substituted for the *HADH2* ** Mental retardation X-linked syndromic 10 [61].

**Table 2:** Generalized alcohol dehydrogenase activities of ABAD/ERAB *.

Substrate or Alcohol	Km	Vmax	kcat ^a^	Catalytic Efficiency kcat/Km
	mM	Units/mg	S^−1^	M^−1^ S^−1^
Reduction of S-acetoacetyl-CoA ^b^	0.068 ± 0.020	430 ± 45	190	2.8 × 10^6^
Oxidation of alcohol substrates ^b^				
17beta-Estradiol	0.014 ± 0.006	23 ± 3	10	7.4 × 10^5^
Methanol	No activity	No activity	No activity	No activity
Ethanol	1210 ± 260	2.2 ± 0.4	1.0	0.82
Isopropanol	150 ± 17	36 ± 2	16	110
n-Propanol	272 ± 62	4.2 ± 0.5	1.9	6.9
n-Butanol	53 ± 6	9.0 ± 0.3	4.0	76
Isobutanol	56 ± 16	8.0 ± 0.7	3.6	64
n-Pentanol	18 ± 5	6.9 ± 0.4	3.1	170
(±)-2-Octanol	85 ± 17	245 ± 20	110	1300
(+)-2-Octanol	84 ± 16	102 ± 8	46	540
(−)-2-Octanol	43 ± 9.0	133 ± 23	60	1400
n-Decanol	14 ± 6.3	2.8 ± 0.5	1.3	90

^a^ Calculation based on 1 unit representing one µmol of product formed per min and a molecular mass of the enzyme as 26,926 Da. ^b^ Experiments were performed by incubating ERAB/HADHII with a range of concentrations of the indicated substrates in the presence of NAD^+^/NADH. Details of experimental procedures are described in the text of Ref. [53]. * This table was reproduced from Table 1 of Ref. [53].

## Data Availability

Not applicable.

## Glossary

ABAD	Aβ-binding protein alcohol dehydrogenase
AD	Alzheimer’s disease
ADH	Alcohol dehydrogenase
BCAT	Branched-chain amino-transferase
BCKDC	Branched-chain alpha keto acid dehydrogenase
COX	Cytochrome C oxidase
ERAB	Endoplasmic reticulum-associated Aβ-binding protein
HAD	l-3-hydroxyacyl dehydrogenase
HADH2	3-hydroxyacyl-CoA dehydrogenase type 2
HBD	Hydroxybutyric acid dehydrogenase
17β-HSD10	17β-hydroxysteroid dehydrogenase type 10
MHBD	Methylhydroxybutyryl-CoA dehydrogenase
MRPP2	Mitochondrial ribonuclease P protein 2
OMIM	Online Mandelian Inheritance in Man (see NIH website)
PDI	Protein disulfide isomerase
SCHAD	Short-chain 3-hydroxyacyl-CoA dehydrogenase
SDR5C1	Short-chain dehydrogenase/reductase 5C1
PD	Parkinson’s disease
TCA	Tricarboxylic acid
VDAC	Voltage-dependent anion channel
